# Innate Immune Evasion of Porcine Epidemic Diarrhea Virus Through miRNA-193a-5p/IL22/pBD1 Pathway in Intestinal Epithelium

**DOI:** 10.1155/tbed/7421187

**Published:** 2025-11-11

**Authors:** Qixian Feng, Jiaqi Chen, Jiancheng Chen, Yu Zheng, Ruisen Wu, Lihui Xu, Longbai Wang, Quanxi Wang

**Affiliations:** ^1^College of Animal Science, Fujian Agriculture and Forestry University, Fuzhou 350002, China; ^2^Fujian Key Laboratory of Traditional Chinese Veterinary Medicine and Animal Health, Fujian Agriculture and Forestry University, Fuzhou 350002, China; ^3^University Key Laboratory for Integrated Chinese Traditional and Western Veterinary Medicine and Animal Healthcare in Fujian Province, Fujian Agriculture and Forestry University, Fuzhou 350002, China; ^4^Institute of Animal Husbandry and Veterinary Medicine, Fujian Academy of Agriculture Sciences / Fujian Animal Disease Control Technology Development Center, Fuzhou 350013, China

**Keywords:** immunologic escape, interleukin 22, miRNA-193a-5p, porcine β-defensin 1, porcine epidemicdiarrhea virus

## Abstract

Porcine epidemic diarrhea (PED) virus (PEDV) is a highly contagious intestinal infection that primarily affects suckling pigs. The interaction about the innate immune evasion of PEDV in intestinal epithelium and microRNA (miRNA) remains unclear. A strain of PEDV belonging to the G2a genotype, designated FJND 2022, was successfully isolated and confirmed. Then, the miRNA profile in exosomes-derived from intestinal porcine epithelial cell line (IPEC) infected with PEDV FJND 2022 for 48 h was evaluated. In exosomes from PEDV-infected IPECs, 34 miRNAs showed differential expression relative to blank cells. A total of 7762 target genes of those differentially expressed miRNAs were forecast, and the miR-193a-5P and its target mRNA interleukin (IL)22 and porcine β-defensin 1 (pBD1) attracted our interest. After infection with PEDV for 48 h, the mRNA levels and protein levels of IL22 and pBD1 were both notably downregulated, while the mRNA level of miR-193a-5P was significantly decreased. When IPECs were pretreated with the mimic of miR-193a-5P and then infected with PEDV, the mRNA levels of IL22 and pBD1 were significantly increased while the viral load of PEDV was significantly reduced. However, siRNA-mediated knockdown of IL22 abrogated the capacity of miR-193a-5p mimic pretreatment to restore pBD1 expression. Furthermore, the inhibitor of miR-193a-5P was pretreated with IPECs infected with PEDV, resulting in a notable downregulation of IL22 and pBD1 expression, and a significant upregulation of the virus load of PEDV. Finally, we also found that the expression levels of IL22, pBD1, and miR-193a-5P were notably reduced in the small intestinal epithelium of suckling piglets infected with PEDV for 48 h. Therefore, in this study we reveal that PEDV downregulates the miR-193a-5P expression in the intestinal epithelium to evade the antivirus of IL22/pBD1, which provides new insights into PEDV molecular pathogenesis and immune evasion mechanisms.

## 1. Introduction

Porcine epidemic diarrhea (PED) virus (PEDV), classified as a member of the *Alphacoronavirus* genus, is recognized as an easily communicable intestinal disease primarily affecting sucking pigs [1]. The PEDV S protein is known to elicit antibody responses. Amino acid variations in its N-terminal domain serve as a basis for defining its classification into two distinct genotypes, G1 and G2. The genogroups can be further categorized into G1a (CV777), G1b, G2a, G2b (Asian strains), and G2c. These PEDV strains exhibit distinct geographic distribution patterns and have been identified globally, including in regions such as the United States, Europe, and China [2, 3]. This highlights the dynamic nature of PEDV evolution. Since its initial identification in China in 2011, G1b has exhibited widespread prevalence across Asia [4]. A G2b strain of PEDV, similar to the strain that has been circulating in China since 2011, was introduced into a region with an intensive pig population in the Midwest of USA and spread rapidly throughout the country [5]. In recent years, despite the widespread availability of PEDV vaccines, they have not succeeded in curbing the global prevalence of PED [6].

Researching the host factors involved in PEDV infection is of great importance for devising new strategies to control its spread. The expression of interferon (IFN) type III was inhibited by PEDV E protein [7]. The report revealed that PEDV infection can promote the upregulation of karyopherin α 2 (KPNA2). KPNA2 bound to and targeted the PEDV E protein for degradation via autophagy, thereby inhibiting PEDV replication [8]. Polypyrimidine tract-binding protein 1 (PTBP1) is an RNA-binding protein that play a crucial role in inhibiting PEDV replication by inducing autophagic protein degradation to inhibit IFN [9]. MicroRNA (miRNA)-328-3p modulates ZO-1 expression, thereby influencing the PLC-β1-PKC pathway, which in turn inhibits the proliferation of PEDV [6]. This regulatory mechanism highlights the role of miRNA-328-3p in viral infection control and suggests potential therapeutic targets for PEDV management. Exosomes not only serve as vehicles for PEDV transmission, but also modulate host immune responses and cellular signaling pathways, further influencing viral pathogenesis [10, 11].

The intestinal epithelium is integral to the innate immunity of the intestines. It establishes chemical and physical barriers that safeguard the host. The diverse secretory cells within the intestinal epithelium are essential for preserving the integrity of the epithelial barrier [12]. Mucus forms a physical barrier and antimicrobial peptides exhibit broad-spectrum antimicrobial activity, primarily secreted by goblet cells and Paneth cells, play critical roles in maintaining intestinal homeostasis [13, 14]. The viscoelastic properties of mucus are crucial for its barrier function. High-molecular-mass mucins are key contributors to the viscoelasticity of mucus, underpinning its effectiveness as a protective barrier [15]. In vertebrates, defensins vary in the peptide length between six cysteines and their disulfide bond connectivity. Based on the specific arrangement of disulfide bonds, they are classified into three subfamilies: α-defensins, β-defensins, and θ-defensins [16]. Beta-defensins, a type of host defense peptide, exhibiting diverse biological functions, including promoting cytokine secretion and regulating inflammatory responses [17]. The porcine β-defensin 1 (pBD1) gene provides defense against *Bordetella pertussis* in newborn piglets, which is crucial for regulating immune responses against respiratory infections. Short-chain fatty acids supplementation induced BD1, which may be involved in inhibiting the inflammatory response and enhancing intestinal barrier integrity in intestinal porcine epithelial cell line (IPEC)-J2 [18].

In this study, we isolated and characterized PEDV strain FJND 2022, and wanted to explored the interaction about the innate immune evasion of PEDV in intestinal epithelium and miRNA, revealed the mechanism of miR-193a-5P in regulating PEDV proliferation to evade intestinal innate immunity.

## 2. Materials and Methods

### 2.1. Animal

The suckling piglets were provided by Fujian Ruoxi Ecological Agriculture Development Co., Ltd, which were tested negative for both the antigen and antibodies of PEDV. These piglets were derived from the Pig Improvement Company (PIC).

### 2.2. Ethics Approval

This study that involved animals was supervised by the Fujian Academy of Agricultural Sciences (IAHV-AEC-2021-066), and was conducted by following the guidelines and protocol.

### 2.3. Primers Synthesis

The specific primers for polymerase chain reaction (PCR) targeting PEDV (AF353511), transmissible gastroenteritis virus ([TGEV] DQ811788), pseudorabies virus ([PRV] JF797217), classical swine fever virus ([CSFV, KC989052), porcine circovirus type 2 ([PCV2] AY035820), porcine reproductive and respiratory syndrome virus ([PRRSV] AY032626), porcine deltacoronavirus ([PDCoV] MH708123), and African swine fever virus ([ASFV] AF302812) were designed using Primer Premier 5.0 (Table 1). These primers were synthesized by Sangon Bioengineering (Shanghai) Co., Ltd.

### 2.4. Isolation and Characterization of PEDV Strain FJND 2022

#### 2.4.1. Collection of Intestinal Tissues

In 2022, a significant number of suckling piglets on a pig farm in Fujian Province, China, experienced severe watery diarrhea, leading to increased mortality. Five samples of the small intestine from the deceased pigs were collected aseptically after removing the intestinal contents.

#### 2.4.2. RNA Extraction, Reverse Transcription, and PCR

RNA was extracted with a RNA extraction kit, following the manufacturer's guidelines. The RNA samples were then reverse transcribed into complementary deoxyribonucleic acid (cDNA) following the instructions provided by the reverse transcription kit. Both kits were obtained from TransGen Biotech in Beijing China. See Table 1 for PCR primers and refer to our previous study [19] for specific conditions.

### 2.5. PEDV Isolation

The intestinal tissues that tested positive for PEDV were washed three times with sterilized normal saline. The intestinal tissues were then ground using sterilized normal saline containing penicillin and streptomycin. After three freeze–thaw cycles, samples were centrifuged at 10,000 rpm for 0.5 h, then filtered through a 0.22 μm membrane and stored at −80°C. Vero-E6 cells were passaged and cultured for virus isolation. As described by Hao et al. [20], the cells were treated with the tissue sample filter at 37 °C for 2 h. Cells were then covered with cell medium containing 2% trypsin for 48 h. The virus was transmitted unknowingly for five generations. The viral load was assessed using PCR. The PCR product was sent for sequencing analysis. The Reed–Muench method [21] was employed in the median tissue culture infective dose (TCID_50_).

### 2.6. Immunofluorescence for PEDV Detection

IPEC-J2 cells were subcultured on cell culture inserts (Thermo Fisher Scientific, Beijing, China) in 24-well plates. After 48 h postinfection with PEDV, the cells were fixed and washed according to the method of Zhang et al. [22]. After blocking with 0.5% BSA for 1 h at 37°C and washing three times with 1× PBS, cells were incubated overnight at 4°C with a primary antibody against the PEDV N protein provided by Dr. Qiuyong Chen. Following three washes, cells were exposed to Cy3-conjugated goat antimouse IgG (H+L) in the dark for 2 h. Cells were then washed again, incubated with DAPI, and assessed using fluorescence microscopy [23].

### 2.7. Ultra Microstructure Observations by Electron Microscopy

IPEC cells infected with PEDV for 48 h were subjected to centrifugation at 10,000 × *g* for 45 min. The cells were resuspended in ultrapure water, initially fixed with 2.5% glutaraldehyde for 2 h, then enhanced with 1% osmic acid, and embedded in epoxy resin. Section size was 55 ± 5 nm. Then, the sections were stained with 3% uranium acetate-citric acid. The viral morphology was examined using an HT7700 electron microscope [21].

### 2.8. Animal Infection With PEDV FJND2022

Six PEDV antigen and antibody double-negative 1-day-old sucking piglets, which were also negative for PCV2, PRRSV, PRV, and CSFV antigens, were equally divided into two groups: an infection group and a control group (Supporting Information 1: Supplement Data S1). In the infection group, the subjects received intramuscular injections and oral administration of 0.5 mL of PEDV (TCID_50_ = 10^−5.5^/0.1 mL) each. The control group was treated with intramuscular injections and oral administration of 0.5 mL of sterilized physiological saline. The health status of all pigs was recorded daily. If the piglets died, they were dissected, and the small intestine was collected for PEDV detection using PCR. Subsequently, hematoxylin–eosin (H and E) staining was performed on the generated pathological sections to observe any pathological alterations.

### 2.9. Relative Expression Levels of Interleukin (IL)22, pBD1, and miR-193a-5p in Small Intestine Tissues

Then, we aimed to determine if miR-193a-5p is regulated during PEDV infection. The expression levels of miR-193a-5p were determined using RT-qPCR with specific primers (miR-193a-5 pF 5′-CGATTATGGGTCTTTGCGGGCG-3′; R 5′-AGTGCAGGGTCCGAGGTATT-3′). GAPDH was used as the reference gene (GAPDH F 5′-CGCCAACATCAAATGGGGTG-3′; R 5′-CACGCCCATCACAAACATGG-3′). The reaction system for RT-qPCR is referenced from our previous study [19]. The relative expression of miRNA was calculated using the 2^−ΔΔCT^ method [24].

### 2.10. Isolation and Identification of Exosomes From IPECs

To investigate the regulatory role of miR-193a-5p from exosomes in PEDV infection, IPECs were subcultured. At 48 h post-PEDV infection, the supernatant of cells from the challenged and control groups was collected. The sample was filtered using a 0.22 μm filter to remove cell debris and other impurities. Subsequently, it was centrifuged at 3000 rpm at 4°C for 20 min. The supernatant was collected and fixed with VEX exosome isolation reagent (Nanjing Vazyme Biotech Co., Ltd). The mixture was stored at 4 °C overnight. The sample was then centrifuged at 10,000 rpm at 4°C for 30 min. The precipitates were resuspended in PBS, and the protein concentration was measured using the BCA method. They were stored at −20 °C.

Then, the exosome sample was fixed with 2.5% glutaraldehyde on the copper grid for 5 min and subsequently stained with a saturated solution of uranyl acetate for 1 min. The samples were washed three times with ddH2O and then observed for exosome morphology using a transmission electron microscope (HT7700).

### 2.11. Western Blot

The total protein of exosome samples, IPEC cells and small intestine tissues of piglets were extracted following the kit instructions (PC0020-500, Beijing Solarbio Science and Technology Co., Ltd). After preparing a 10% resolving gel and a 5% stacking gel, the total proteins were separated at 90 V for 40 min using sodium dodecyl sulfate polyacrylamide gel electrophoresis (SDS-PAGE). The polyvinylidene difluoride ([PVDF], 0.22 μm, ISEQ00010), which had had been pretreated with transfer buffer at 4°C, was used to transfer the proteins under a voltage of 100 V for 90 min. After treating the PVDF membrane with sealing fluid for 0.5 h, the exosome samples were then incubated with primary antibodies against TSG101 (1:2000, 28283-1-AP), CD9 (1:1000, 20597-1-AP), or calnexin (1:5000, 10427-2-AP). Concurrently, IPEC cells and small intestine tissues were incubated with primary antibodies specific to IL22 (1:5000, ER1803-85) and defensin β1 (1:1000, bs-1163R). All samples were then conjugated with horseradish peroxidase (HRP) and incubated at 4°C overnight. Then, they were incubated with the rabbit antipig IgG secondary antibody at room temperature for 2 h. Immunoblots were developed using the enhanced chemiluminescence reaction.

### 2.12. miRNA Profile of Exosomes From IPECs

To investigate whether the miRNAs were derived from exosomes, the exosomes from IPECs that were treated with PEDV or control, were sent to Shanghai Biotechnology Corporation (Shanghai, China) for miRNA sequencing by Illumina NovaSeq6000 S4 PE150 (California, USA). The sequences containing the adaptors or length less than 10 bp in the draw data were filtered out, which were clean reads. For subsequent statistical analysis, clean reads with lengths ranging from 18 to 40 nt were selected.

Each miRNA underwent normalization via the trimmed mean of *M*-values method and was subsequently converted into transcripts per million. The differential expression miRNAs were analyzed by edge R package, and the *p*-value were corrected to *q*-value through multiple hypothesis testing. The *p*-value threshold was calculated using the false discovery rate (FDR) method. Also, the differential expression fold, namely fold-change. The differential expression miRNAs were: *q*-value ≤ 0.05, fold-change ≥ 2 [25, 26]. The targeted binding mRNA of the differential expression miRNA (including known miRNA and novel miRNA) was predicted online (http://www.microrna.org/microrna/home.do).

### 2.13. Reconfirmation by RT-qPCR

At 48 h postinfection, the supernatant from the cells was collected. RNA was denatured and melted, consisting of 1 μL of RNA, 0.5 μL of miR-193a-5pF primer, 0.5 μL of R primer, and 13 μL of RNase-free ddH_2_O (primers were shown in Table 2). The reaction was carried out at 65 and 4°C for 5 min, respectively. Then, reverse transcription procedure refers to Xiang's study [18]. These six differentially expressed miRNAs had their expression levels reconfirmed by RT-qPCR. The viral load of PEDV was determined using RT-qPCR with a standard curve equation (*y* = −3.3863*x* + 44.734, *R*^2^ = 0.9891).

### 2.14. miR-193a-5P Mimic/Inhibitor Treated With IPECs

The miR-193a-5p and its targeted mRNA IL22 and pBD1 caught our attention. IPECs were subcultured in 6-well cell culture plates with DMEM medium containing 10% exosome-free FBS. The mimic (Zolgene Biotech, Fuzhou, China) or inhibitor (Zolgene Biotech, Fuzhou, China) of miR-193a-5p was transfected into IPECs using lipofectamine 3000. At 24 h post-transfection, cells were treated with PEDV at a multiplicity of infection (MOI) of 1 for 48 h in the DMEM medium supplemented with 2% pancreatin. Cells were treated only with a mimic or PEDV as a control. Cells were not treated with mimic and PEDV, serving as the mock control. At 48 h post PEDV infection, exosomes were extracted from cells, and the relative expression levels of miR-193a-5p in each group were determined by RT-qPCR. Then the relative mRNA expression levels of IL22 (F: 5′´-TTCCAGCAGCCCTACATCAC-3′; R:5′-GTAGCAGCGCTCTCTCATCT-3′) and pBD1(F:5′-GGCAAGTGTGCTCCAAAGATG-3′; R:5′-ATGCTTTTCCAAGGGAGCAG-3′) in each group were evaluated by RT-qPCR, respectively. The viral load of PEDV was determined by RT-qPCR with standard curve (*y* = −3.3863*x* + 44.734, *R*^2^ = 0.9891).

### 2.15. Silent Expression of IL22 in IPECs

Three siRNA-IL22 pairs, designed and synthesized by Fuzhou Zolgene Biotech Co. Ltd as follows: (1) F: 5′-CUUUAAAGACACAGUGAAATT-3′, R: 5′-UUUCACUGUGUCUUUAAAGTT-3′; (2) F: 5′-GAGGCUAGUUUGGCAGAUA TT-3′, R: 5′-UAUCUGCCAAACUAGCCUCTT-3′; (3) F: 5′-CUGGACAGCCU CAGCAAAATT-3′, R: 5′-UUUUGCUGAGGCUGUCCAGTT-3′. These pairs of siRNA-IL22 (5 µg) were transfected into IPECs using Lipofectamine 3000. At 24 h post-transfection, RT-qPCR measured IL22 mRNA levels to pick out the sequence with the best RNA interference. IPECs were subcultured in 6-well cell culture plates with the DMEM medium. At 6 h post-transfection with 5 µg of siRNA-IL22, cells were treated with one MOI of PEDV for 48 h. Then, RT-qPCR quantified miR-193a-5p, IL22, and pBD1 expression and PEDV viral load, with a standard curve for quantification.

### 2.16. Coimmunprecipitation (co-IP) Analysis

Small intestinal tissue homogenates were mixed with 10 volumes (v/w) of IP lysis buffer (G2038-100 ML, Servicebio) and mechanically homogenized. After thorough vortexing, 20 μL of SweMagrose Protein G magnetic beads were added, followed by end-over-end rotation at 4°C for 1 h. The mixture was centrifuged at 2000 × *g* (4°C, 5 min), and the supernatant was divided equally into two 1.5 mL microcentrifuge tubes. For the IP group, an IP-grade antibody was added to one aliquot. The IgG control group received species-matched normal IgG at equivalent concentration. Both samples were incubated overnight at 4°C. Subsequently, 100 μL of SweMagrose Protein G magnetic beads were added to each tube, with continuous rotation at 4°C for 2–4 h. After magnetic rack immobilization (1 min), supernatants were carefully aspirated to obtain immunocomplexes. Beads were washed five times with 500 μL ice-cold TBS buffer. Immunoprecipitated complexes were resuspended in 100 μL 1× reducing Laemmli buffer, denatured at 95°C for 10 min in a metal bath, and centrifuged at 1000 × *g* (4°C, 5 min). Resultant supernatants were subjected to WB analysis. The immunoprecipitation-grade antibodies used were: anti-IL22 (Rab (P) 13444) and antidefensin beta 1 (Rab (P)12892).

### 2.17. Statistical Analysis

The data were showed as the mean ± standard difference (SD) unless noted otherwise, and they were analyzed by *t*-test or independent-samples ANOVA using the SPSS statistical software (Ver.16.0 for windows, SPSS Inc., Chicago, U.S.A). *p* < 0.05 was considered statistically significant.

## 3. Result

### 3.1. Isolation and Identification of PEDV Strain FJND2022

**Figure 1:**
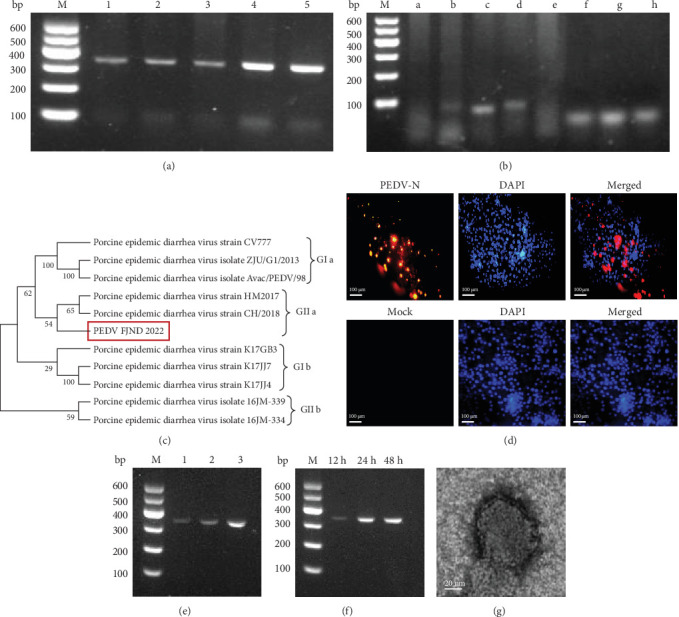
Isolation and identification of PEDV strain FJ2022. (A) PCR identification for PEDV (lane M: 600 DNA marker; lane 1–5: random site detection of diseased materials). (B) PCR amplification for virus in diseased materials (lane M: 600 DNA marker; lane a: TGEV primer; lane b: PoRV primer; lane c: CSFV primer; lane d: PRV primer; lane e: PDCoV primer; lane f: PCV2 primer; lane g: ASFV primer; lane h: PRRSV primer). (C) Phylogenetic tree of PEDV S genes. The phylogenetic analysis by MEGA 7.0 software program. (D) IPECs were incubated with PEDV N protein monoclonal antibody Cy3-labeled goat antimouse IgG. Nuclei were stained by DAPI (40×). (E) PCR amplification of PEDV for different generations of cells on the diseased material (lane M: 600 DNA marker; lane 1–3: first to third generation of cells on the diseased material). (F) The third generation of viruses infected IPECs for 12, 24, and 48 h. (G) TEM image of PEDV FJ2022 virus. Scale bar is 20 nm.

Five intestinal samples were detected using PCR with PEDV M gene specific primers. The results of agarose gel electrophoresis showed a distinct band in each lane, indicating the presence of the PEDV M gene at approximately 315 bp (Figure 1A). This suggests that the diseased pigs were indeed infected with PEDV. The PCR production from the fourth sample was the brightest. Afterward, the fourth sample was analyzed using PCR. The results indicated that TGEV, PoRV, CSFV, PRV, PDCoV, PCV2, ASFV, and PRRSV were all negative, only PEDV was positive (Figure 1B). This finding suggests that the sick pig was solely infected with PEDV. Then the PCR amplicons preformed with primers basing M gene underwent sequencing, and the subsequent homology analysis indicated that the PEDV strain is classified under G2a. (G2a, Figure 1C).

Furthermore, PEDV was isolated from the IPEC-J2 cell line. Viruses were subcultured three times in the cells. The isolated PEDV was named strain FJND2022. At 48 h postinfection with PEDV strain FJND2022, the cells exhibited noticeable lesions and significant fluorescence when observed under a fluorescence microscope using an antibody against the PEDV N protein (Figure 1D). The virus load was determined by PCR. The results showed that in the three generations of the virus, the band of PCR production from the third generation was the brightest, indicating active viral replication (Figure 1E). The third generation of viruses infected IPECs for 12, 24, and 48 h. The bands of PCR production from the second and third lanes were brighter than those from the first lane. This indicated that PEDV FJND 2022 infected IPECs for 24 and 48 h, resulting in a high viral load (Figure 1F). The size of the PEDV FJND 2022 virus was approximately 140 nm, and its morphology appeared round and capsular under an electron microscope (Figure 1G). The results above revealed that PEDV FJND 2022 were isolated.

### 3.2. Artificial Infection of Piglets With PEDV FJND 2022

**Figure 2:**
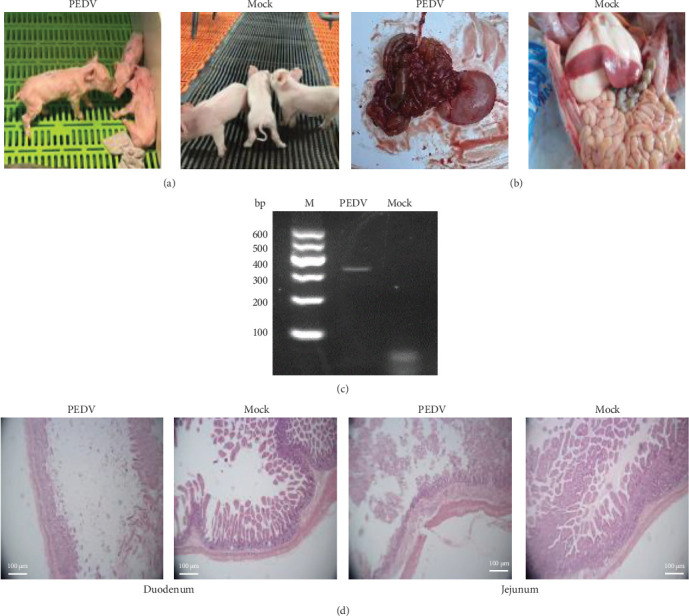
Artificial infection of piglets with PEDV strain FJND-2022. (A) The antibody and antigen of PEDV double negative suckling piglets were artificially infected with PEDV FJ2022 for 5 days (oral administration), and the mock group received DMEM orally. (B) Images of infected and uninfected piglets dissected after 5 days. (C) PEDV M gene amplification in the intestine of infected piglets. (D) H and E staining of the duodenum and jejunum. The scale bar is 100 µm.

The antibody and antigen of PEDV double negative suckling piglets were artificially infected with PEDV FJND 2022 for 5 days, the infected piglets showed severe diarrhea (Figure 2A,B), the stomach and intestines had severe flatulence and bleeding, and two of total three piglets died. The intestinal tract of the piglets was dissected to observe any pathological changes. RNA was extracted from the intestinal tract, and the PEDV S1 gene was successfully amplified, while no other common diarrhea viruses were detected (Figure 2C). The results showed that the PEDV virus had been successfully isolated from the diseased samples, and oral administration of PEDV could induce sickness in piglets. According to H and E staining results of the sections, the duodenal lesions in piglets from the challenge group were more pronounced compared with those in the negative control group. The duodenal villi exhibited high levels of atrophy, distinct fractures, necrosis, and even exfoliation, along with a slight congestion in capillaries (Figure 2D). The number of intestinal villi in the jejunum was reduced, and the reduction was slightly less pronounced than that in the duodenum. The villi in the jejunum were poorly defined and obviously broken, indicating more serious damage.

### 3.3. Exosomes Induced by PEDV From IPECs

**Figure 3:**
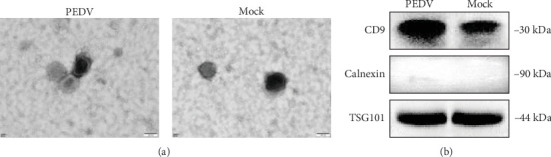
Exosomes-derived from IPECs infected with PEDV. (A) TEM images of exosomes derived from PEDV-infected IPECs and mock groups. (B) Western blotting was performed using the primary antibody of exosomal markers CD9, calnexin, and TSG101.

Exosomes are the important molecular structure for information transfer between cells. To explore whether exosomes were induced from IPECs infected by PEDV. At post 48 h infection with PEDV, the exosomes were isolated. Utilizing transmission electron microscopy (TEM), we observed a distinct morphological alteration in the vesicles emanating from IPECs post PEDV infection; these vesicles, characterized by their size and shape, were consistent with the typical features of exosomes and demonstrated an increase in quantity relative to the mock group (Figure 3A). The protein of the exosome was corroborated by BCA assay, revealing an upregulation in subsequent to PEDV infection (from 1.56 to 1.66 μg/μL). In addition, results of Western blotting using commonly used exosome marker TSG101 and CD9 antibody as well as exosome protein negative marker calnexin indicated successful exosome extraction (Figure 3B). Collectively, these findings underscore the notion that the IPEC cells, upon encountering the PEDV FJND 2022, are prompted to engage in the active secretion of exosomes, thereby potentially modulating intercellular signaling pathways in the context of viral infection.

### 3.4. Different Expression Profile of miRNA in Exosomes From IPECs Infected With PEDV

**Figure 4:**
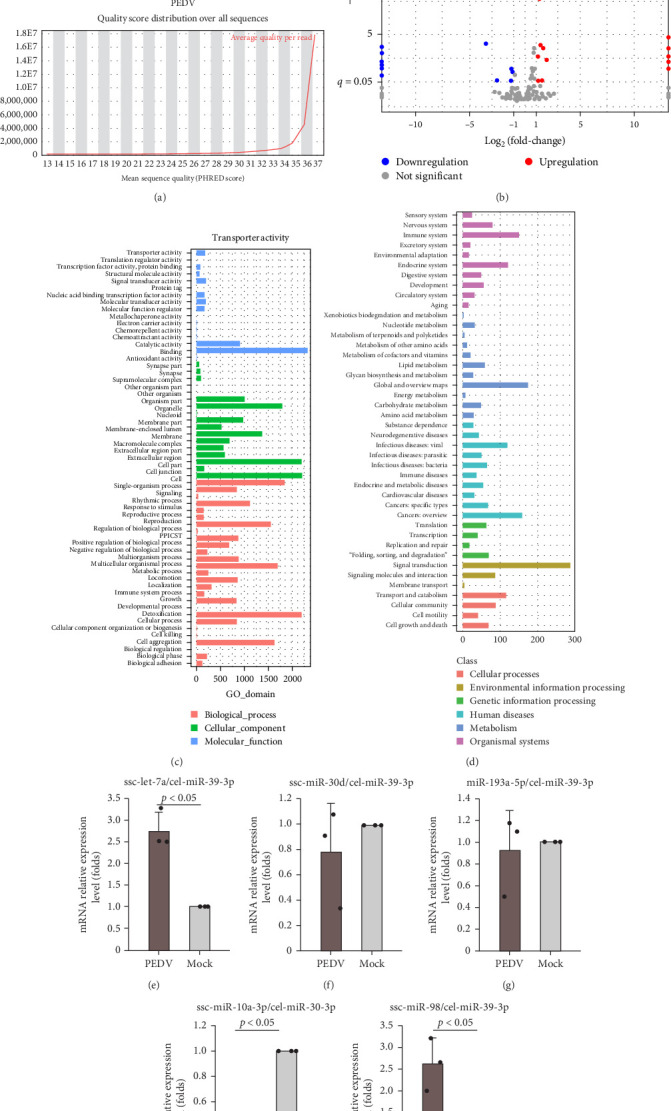
Different expression profile of microRNA in exosomes from IPECs. (A) PHRED quality scores for reads in the PEDV infection and mock groups. (B) Volcano diagram of differential expression of miRNAs. (C) GO functional classification statistics of differentially expressed miRNA target genes. (D) KEGG pathway classification statistics of differentially expressed miRNA target genes. The mRNA levels of ssc-let-7a (E), miR-30d (F), miR-193a-5p (G), miR-10a-3p (H), and ssc-miR-98 (I) were determined by RT-qPCR.

Furthermore, we sent the exosomes from IPECs for miRNA sequencing to confirm which miRNAs play a key role during the PEDV FJND 2022 infection. The reads quality from two groups were present in Figure 4A, and the total number of miRNAs was 463, including 427 known miRNA and 36 novel miRNAs (Supporting Information 2: Supplement Data S2). The total number of different expressions miRNA was 224, including 19 significant down expression and 15 significant up expression (Figure 4B). Total 7762 target genes of all significantly different expression miRNAs were present in Supporting Information 3: Supplement Data S3. Results of target genes GO functional analysis showed that target genes took part in three GO domains as biological_process, cellular_component, and molecular (Figure 4C), in which most target genes involved in binding, cell, and detoxification functions. Target genes involved in KEGG function were analyzed, results indicated that they involved in immune system, endocrine system, infectious diseases: viral, and signal transduction, etc. (Figure 4D), and involved in 273 KEGG pathway (Supporting Information 4: Supplement Data S4). RT-qPCR validated the significantly different miRNA expression levels, with results corroborating the sequencing data (Figure 4E–I), which confirms the reliability of the miRNA sequencing.

### 3.5. PEDV Downregulated miR-193a-5P, IL22, and pBD1 Expression

**Figure 5:**
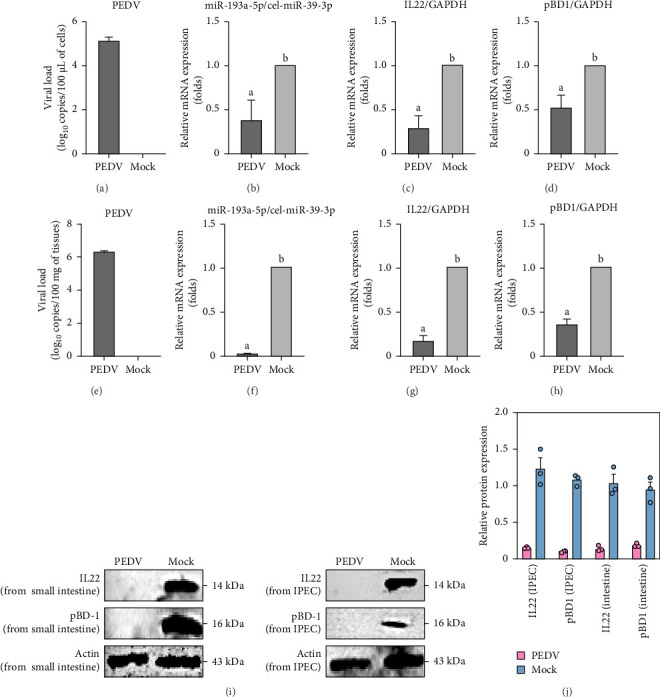
PEDV downregulates miR-193a-5p in IPECs to evade the antiviral effect of IL22/pBP1. At 48 h postinfection with PEDV (A), the mRNA levels of miR-193a-5p (B), IL22 (C), and pBD-1 (D) in IPECs were determined by RT-qPCR. Then at 48 h postinfection with PEDV in the small intestine epithelium of piglets (E), the mRNA levels of miR-193a-5p (F), IL22 (G), and pBD-1 (H) were detected by RT-qPCR. (I) IL22/pBD-1 protein levels were examined by Western blotting in PEDV-infected piglets or IPEC J2. (J) Image J quantification of Western blot band intensities. The a and b bars in each panel without a common superscript letter were signifcantly different (*p* < 0.05).

The miR-93a-5p attracted our interest. When PEDV infected IPECs for 48 h, the expression level of miRNA-193a-5p was significantly downregulated (Figure 5B). Then we evaluated its target gene IL22, and found that the mRNA levels of IL22 and pBD1 were significantly downregulated, too (Figure 5C,D). The above results were consistent with the sequencing result data in IPECs and RT-qPCR results in piglets' data (Figure 5).

In addition, our Western blot results revealed that the degradation of IL22 and pBD1 proteins occurred in IPEC and piglet small intestine following PEDV infection (Figure 5I,J).

### 3.6. PEDV Downregulated miR-193a-5P/IL22/PBD1 Pathway to Promote Its Multiplication

**Figure 6:**
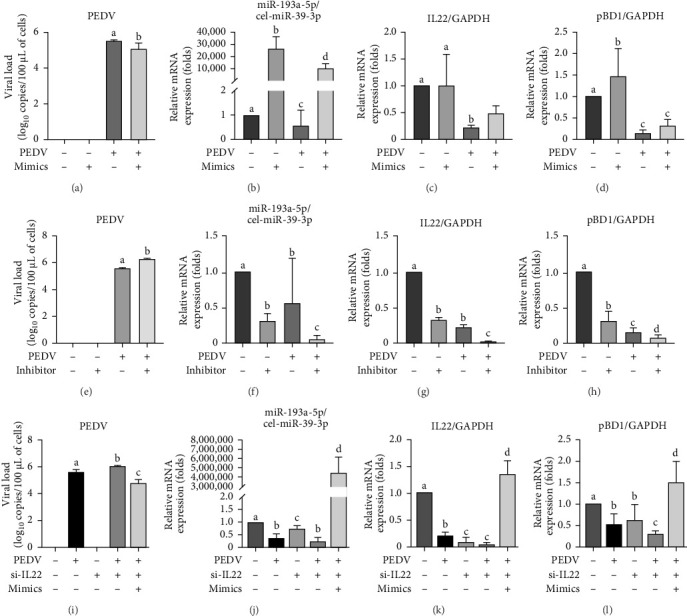
PEDV downregulated miR-193a-5P/IL22/PBD1 pathway to promote its multiplication. Effect of miR-193a-5p mimic on mRNA expression of PEDV (A), miR-193a-5p (B), IL22 (C), and pBD-1 (D) in IPECs. Effect of miR-193a-5p inhibitor on mRNA expression of PEDV (E), miR-193a-5p (F), IL22 (G), and pBD-1 (H) in IPECs. Effect of small interfering IL22 on mRNA expression of PEDV (I), miR-193a-5p (J), IL22 (K), and pBD-1 (L) in IPECs. The a, b, c, and d bars in each panel without a common superscript letter were signifcantly different (*p* < 0.05).

**Figure 7:**
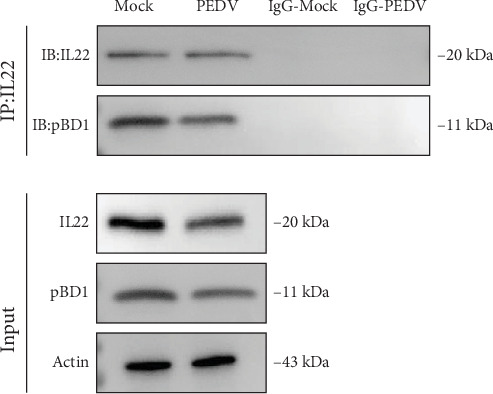
Co-IP analysis of IL22 and pBD1 in small intestine of piglet.

**Figure 8:**
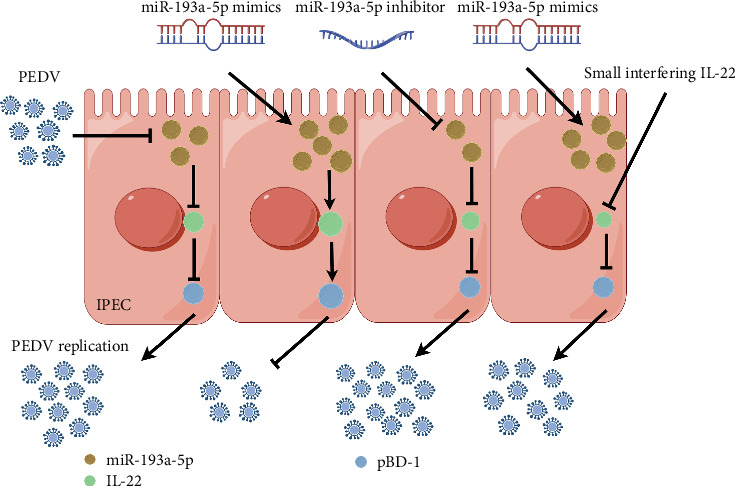
Scheme of PEDV downregulates miR-193a-5p in exosomes from IPECs to immunologic escape from IL22/β-defensin 1 antivirus (by Figdraw).

To investigate PEDV evasion mechanisms against IL22/pBD1-mediated antiviral immunity, we demonstrated that pretransfection of IPECs with the miR-193a-5P mimic prior to PEDV challenge. This pretreatment significantly upregulated IL22 and pBD1 transcript levels and concurrently suppressed PEDV replication (Figure 6). Then the inhibitor of miR-193a-5P pretreated with IPECs that were infected with PEDV, which significantly decreased the mRNA levels of IL22 and pBD1, and promoted the virus load of PEDV FJND 2022 (Figure 6). Furthermore, the mRNA expression of IL22 was inhibited by si-IL22, the miRNA mimic pretreatment cannot recover the mRNA levels of pBD1 (Figure 6). The interactions between IL22 and pBD1 were validated by co-IP analysis (Figure 7). These results revealed that PEDV downregulated miRNA-193a-5p/IL22/pBD1 pathway to immunologic escape from IL22/pBD1 antivirus and to promote its multiplication in porcine intestinal epithelial cells (Figure 8).

## 4. Discussion

In 1984, PEDV was firstly isolated and identified in China [27]. Since 2010, highly virulent PEDV variants have triggered extensive PED outbreaks in China, spreading rapidly across the nation [28, 29]. Now, PEDV has been subdivided into five genotypes. In this study, a PEDV strain designated FJND 2022, was isolated from a pig farm experiencing a PED outbreak. Genetic analysis revealed that this isolate belongs to the G2a genotype, which has been associated with recent PED outbreaks in the region. The identification and characterization of this strain contribute to our understanding of the genetic diversity and epidemiology of PEDV, particularly in the context of ongoing disease surveillance and control efforts. Further analysis of the FJND 2022 strain may provide insights into the pathogenicity and transmission dynamics of PEDV, aiding in the development of targeted intervention strategies to mitigate the impact of PED in swine populations. Vaccination is widely used across the globe for preventing PEDV infection, but it cannot completely prevent PEDV infection because of its genetic variants [30].

Exosomes are usually secreted by all cells, and released into varieties of body fluid [31]. Recently, exosomes play a pivotal role in modulating immune responses, a function that has garnered increasing attention from scholars [28]. Tumor-derived exosomes, which carry immunosuppressive molecules, can suppress cytotoxic immune cells such as CD8+ T cells and natural killer cells [32]. In addition, exosomes carrying minor histocompatibility complex (MHC)–peptide complexes and antigens exhibit the ability to initiate and amplify immune responses [33]. B-lymphoblast-derived exosomes carrying MHC class II-peptide complexes are instrumental in immune responses by activating antigen-specific T cell clones in both humans and mice [34]. This suggests the importance of exosomes in immune regulation. Additionally, exosomes facilitate cell-to-cell viral transmission. The E/NS1 protein, a form of the Zika virus nonstructural protein NS1, has gained attention for its role in immune evasion. Monocytes infected with the Zika virus can carry viral RNA and the E/NS1 protein, thereby promoting viral spread, infection, as well as cell differentiation and activation [35]. Porcine hemagglutinating encephalomyelitis virus (PHEV) is often transmitted via the respiratory route, causing encephalomyelitis in pigs. Furthermore, exosomes derived from PHEV-infected neural cells (PHEV-exos), carry viral RNA and proteins, inducing an innate immune response in nonpermissive bystander cells without requiring immune system involvement [36]. While our study demonstrates that exosomes derived from PEDV-infected IPECs facilitate viral proliferation, strongly associated with downregulation of exosomal miR-193a-5p, it should be noted that observed functional effects may involve synergistic actions of other vesicular constituents.

miR-193a-5p, acting as a transcriptional regulator, is closely linked to inflammatory responses in various diseases through its upregulation or downregulation, and it plays a crucial role in modulating inflammatory mediators [37]. In addition, Feng et al. [38] reported that Qingjie Huagong decoction could enhance MPC-83 cell viability by modulating circHipk3, which functions as a sponge for miR-193a-5p, targeting the NOD-like receptor family pyrin domain-containing 3 (NLRP3) and suppressing pyroptosis-related factors. These findings highlight the potential therapeutic effects of Qingjie Huagong decoction and specific miRNAs in modulating inflammatory responses and cell survival. Another study [39] found that miR-320-5p and miR-193a-5p play a protective role in safeguarding AR42J cells from caerulein-induced injury. These miRNAs achieve their protective effects by targeting the tumor necrosis factor receptor, and miR-193a-5p also can regulate polyunsaturated fatty acids metabolism in bovine mammary epithelial cells by targeting fatty acid desaturase 1 [40]. This study revealed that miR-193a-5p has perfect protective function of epithelia. In our current study, we revealed that PEDV infected in vivo and in vitro downregulated miR-193a-5p expression in exosomes to escape from IL22 and pBD1 antivirus. Notably, miR-193a-5p has been found to play diverse roles in different biological contexts, but how the miRNA was downregulated need to be further research. Furthermore, in the present study, the expression levels of miR-98 and let-7a were significantly upregulated in exosomes derived from IPEC infected with PEDV. Keske et al. [41] reported that miR-98 expression was significantly downregulated in an Aβ42-induced cellular model of Alzheimer's disease. Let-7a functions as a protective miRNA within the gut-heart axis [42]. Its expression is regulated by sex hormones and inflammatory status, and restoring its function may represent a novel strategy for mitigating inflammatory bowel disease (IBD)-associated cardiac complications.

Intestinal mucosal barrier presents immune and microbial barriers that protect the host [43]. Veldhuizen et al. [44] revealed that *Salmonella* serovar could elicit a defense response in the host against bacterial infection, and specific upregulate porcine defensins in a jejunal epithelial cell line. Extracts from *Artemisia annua* enhance the expression of beta-defensins, mucins, and peroxisome-proliferator-activated receptors (PPAR)-γ, facilitating inflammation resolution and the innate immune response [45]. Luo et al. [46] reported that supplementing with 0.1% dietary synbiotics can mitigate inflammatory responses and protect the intestinal barrier by enhancing innate immune function and reducing PEDV genomic copy levels. Beta-defensins can reduce inflammation by specifically binding to microbe-associated molecular patterns and regulate the chemotactic activity of various innate immune cells [47]. Elahi's [48] research shows that pBD-1 is essential for protecting against *Bordetella pertussis* infection. It not only has direct antibacterial activity but also enhances innate immunity. Newborn piglets deficient in pBD-1 are more susceptible to *Bordetella pertussis*. The pBD1 is pivotal role to regulate innate immune responses to combat infections [49]. In this study, our results indicated that PEDV infection could inhibit pBD1 expression. Therefore, improving the intestinal mucosal barrier function of piglets would prevent damage of PEDV to intestinal tract.

## 5. Conclusion

In this study, our team isolated and identified PEDV strain FJND-2022, and firstly reported that PEDV downregulated miR-193a-5P to immunologic escape from IL22/pBD1 antivirus. These results will provide an important complement to the molecular pathogenesis of PEDV infection.

## Figures and Tables

**Table 1:** Primer sequences.

Gene names	Sequences (5′-3′)	Size/bp
PEDV-M-F	TATGGCTTGCATCACTCTTA	315
PEDV-M-R	TTGACTGAACGACCAACACG	
PEDV-S1-F	TTATGAGCCAACTCAAGTGTT	610
PEDV-S1-R	AACACCTGCCAAAAAGC	
TGEV-N-F	TTACAAACTCGCTATCGCATGG	528
TGEV-N-R	TCTTGTCACATCACCTTTACCTGC	
PoRV-F	CCCCGGTATTGAATATACCACAGT	333
PoRV-R	TTTCTGTTGGCCACCCTTTAG	
CSFV-F	AGGAATACAACCACGATTTGCAACT	113
CSFV-R	TGACGCCAAATACCTCCTACTGA	
PRV-F	CTGGCTCTGCGTGCTGTG	348
PRV-R	GGTCCATTCGTCACTTCCG	
PDCoV-F	ATTCTGCTTTGGCTGCTC	359
PDCoV-R	TCCTGTGGCGGATTTC	
PCV2-F	GGGCCAGAATTCAACCTTAACC	171
PCV2-R	CGCACCTTCGGATATACTGTCA	
ASFV-F	AGCTCTTCCAGACGCATGTTCATC	239
ASFV-R	GGTATTCCTCCCGTGGCTTCAAAG	
PRRS-F	GAGTTTCAGCGGAACAATGG	450
PRRS-R	GCCGTTGACCGTAGTGGAG	

**Table 2:** Primer sequences for RT-qPCR.

miRNA names	Position	Sequences (5′-3′)
cel-miR-39-3p	Forward	TCGCGTCACCGGGTGTAAATC
cel-miR-39-3p	Reverse	AGTGCAGGGTCCGAGGTATT
cel-miR-39-3p	RT	GTCGTATCCAGTGCAGGGTCCGAGGT
		ATTCGCACTGGATACGACCAAGCT
ssc-miR-193a-5p	Forward	CGATTATGGGTCTTTGCGGGCG
ssc-miR-193a-5p	Reverse	AGTGCAGGGTCCGAGGTATT
ssc-miR-193a-5p	RT	GTCGTATCCAGTGCAGGGTCCGAGGT
		ATTCGCACTGGATACGACTCATCT
ssc-let-7a	Forward	TCCGCGTGAGGTAGTAGGTTGT
ssc-let-7a	Reverse	AGTGCAGGGTCCGAGGTATT
ssc-let-7a	RT	GTCGTATCCAGTGCAGGGTCCGAGGT
		ATTCGCACTGGATACGACAACTAT
ssc-miR-10a-3p	Forward	GCTGCCGCGCAAATTCGTATCTAG
ssc-miR-10a-3p	Reverse	AGTGCAGGGTCCGAGGTATT
ssc-miR-10a-3p	RT	GTCGTATCCAGTGCAGGGTCCGAGGT
		ATTCGCACTGGATACGACATTCCC
ssc-miR-30d	Forward	GCCGTGTAAACATCCCCGACTG
ssc-miR-30d	Reverse	AGTGCAGGGTCCGAGGTATT
ssc-miR-30d	RT	GTCGTATCCAGTGCAGGGTCCGAGGT
		ATTCGCACTGGATACGACAGCTTC
ssc-miR-98	Forward	GCCGCGTGAGGTAGTAAGTTGT
ssc-miR-98	Reverse	AGTGCAGGGTCCGAGGTATT
ssc-miR-98	RT	GTCGTATCCAGTGCAGGGTCCGAGGT
		ATTCGCACTGGATACGACAACAAT

## Data Availability

The raw sequencing data from this experiment can be accessed via the following link: https://www.ncbi.nlm.nih.gov/bioproject/PRJNA1102139.

## Supporting Information

Supporting Information 1 Supplement Data S1: Six PEDV antigen and antibody double-negative 1-day-old sucking piglets. (A) Porcine epidemic diarrhea virus (PEDV) ELISA kits, model MM-33787O1, from Jiangsu Meimian Industrial Co., Ltd, were used to detect piglet serums. (B) PEDV was amplified from the serum of six piglets.

Supporting Information 2 Supplement Data S2: In Supplement Data S2, we obtained information on a total of 463 miRNAs in exosomes derived from IPEC, of which 427 were known and 36 were novel miRNAs. The title “Supplement Data S2” refers to the file containing the total number of miRNAs obtained. Files starting with “Known” represent the known miRNAs, while those starting with “mock” and “PEDV” correspond to the information of novel miRNAs in the two respective groups.

Supporting Information 3 Supplement Data S3: The miRNA profile in exosomes-derived from IPEC infected with PEDV FJND 2022 for 48 h was evaluated. Total of 7762 target genes of those differentially expressed miRNAs were predicted in Supplement Data S3.

Supporting Information 4 Supplement Data S4: We analyzed the target genes involved in KEGG functions, and the detailed information of 273 KEGG pathways is provided in Supplement Data S4.
