# Supplementary material for: Innate Immune Evasion of Porcine Epidemic Diarrhea Virus Through miRNA-193a-5p/IL22/pBD1 Pathway in Intestinal Epithelium
Source: Transbound Emerg Dis. 2025 Nov 11;2025:7421187. doi: 10.1155/tbed/7421187 (PMC12626695; doi:10.1155/tbed/7421187)
Supplement: Supporting Information 1 — Supplement Data S1: Six PEDV antigen and antibody double-negative 1-day-old sucking piglets. (A) Porcine epidemic diarrhea virus (PEDV) ELISA kits, model MM-33787O1, from Jiangsu Meimian Industrial Co., Ltd, were used to detect piglet serums. (B) PEDV was amplified from the serum of six piglets. [file 7421187.f1.docx]

Supplemental material for **Innate immune evasion of Porcine epidemic diarrhea virus through miRNA-193a-5p/IL22/pBD1 pathway in intestinal epithelium** by Qixian Feng et al. in *Transboundary and Emerging Diseases*


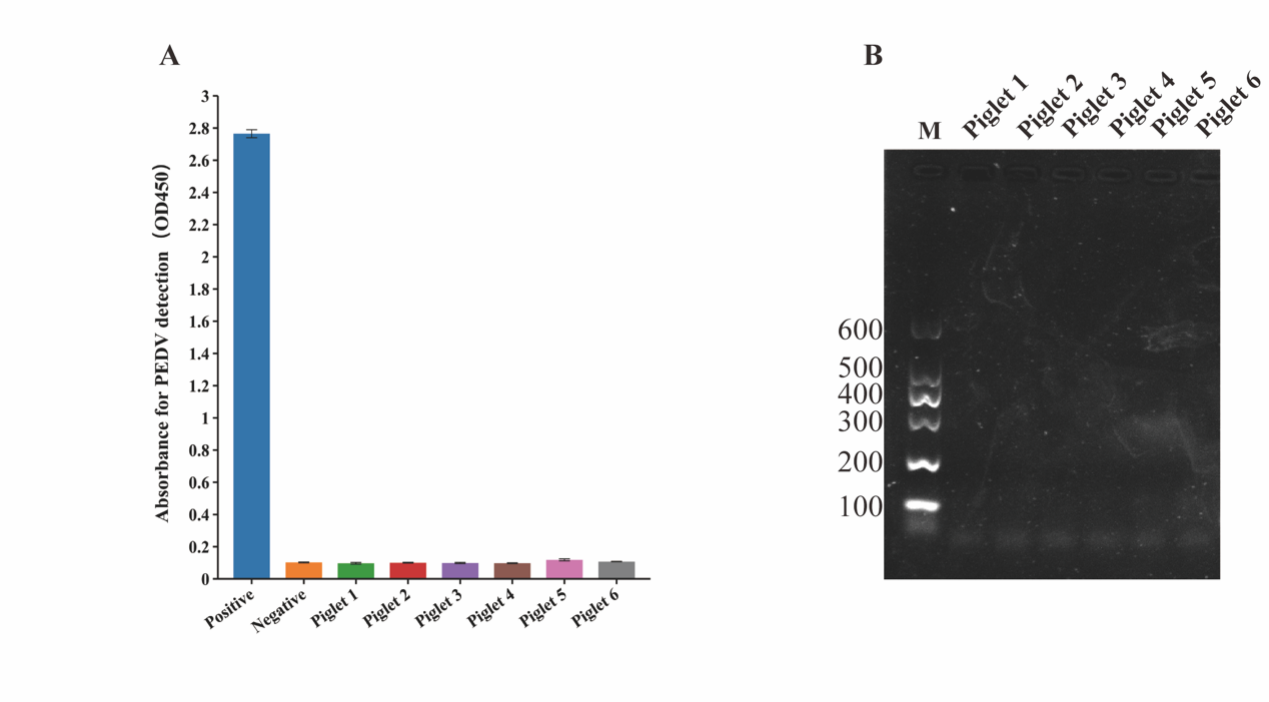


Figure S1 Six PEDV antigen and antibody double-negative 1-day-old sucking piglets. (A) Porcine epidemic diarrhea virus (PEDV) ELISA kits, model MM-33787O1, from Jiangsu Meimian Industrial Co., Ltd, were used to detect piglet serums. (B) PEDV was amplified from the serum of six piglets.
